# Decomposing cognitive-motor planning from execution: smartphone motor sequencing provides scalable digital biomarkers of cognitive-motor function across central nervous system disorders

**DOI:** 10.64898/2026.07.30.26359238

**Published:** 2026-08-02

**Authors:** Peter Kosa, Amir Moghadam Ahmadi, Marie Kanu, Yolanda Mejia, Emmanuel Mekasha, Caledonia Steltzner, Bibiana Bielekova

## Abstract

Dynamic praxis, defined as the ability to plan and execute complex, ordered motor actions, underpins essential activities of daily living and occupational performance. Because motor sequencing depends on distributed frontostriatal and interhemispheric networks, its impairment serves as a sensitive indicator of central nervous system (CNS) dysfunction, yet traditional bedside assessments lack granular subprocess resolution. Here, we digitized Luria’s fist-edge-palm paradigm into a self-administered smartphone task within the Neurological Functional Test Suite (NeuFun-TS) and evaluated its clinical validity in 296 participants (34 healthy donors, 208 people with multiple sclerosis (MS), and 54 neurological controls). We extracted seven digital biomarkers across speed, execution, accuracy, and integrative throughput. Six biomarkers significantly differentiated disease cohorts along an ordinal severity gradient (healthy donors < relapsing-remitting MS < progressive MS), with Motor Sequencing Deficit showing the strongest group separation (r=0.73, p=1.1×10 ^-21^ ). Kinematic decomposition dissociated cognitive planning latency (ε ^2^ =0.011) from pure motor execution (ε ^2^ =0.716). Speed-accuracy tradeoff analysis differentiated secondary-progressive MS (84% slow-and-inaccurate) from primary-progressive MS (19% slow-but-accurate compensatory phenotype). A parsimonious two-biomarker composite achieved high diagnostic classification accuracy (concordance index = 0.868; validation intraclass correlation coefficient ICC = 0.77) and correlated strongly with CNS tissue destruction on MRI (rho=0.29-0.38), clinician disability scales (rho=0.44-0.52), and cognitive performance (rho=0.49-0.61). By capturing subtle cognitive-motor planning and execution deficits, smartphone-based motor sequencing offers a scalable, low-burden framework for longitudinal neurological monitoring in MS and broader central nervous system disorders affecting daily functional independence.

## Full Text Availability

The license terms selected by the author(s) for this preprint version do not permit archiving in PMC. The full text is available from the preprint server.

